# Immunoglobulin G genetic variation can confound assessment of antibody levels via altered binding to detection reagents

**DOI:** 10.1002/cti2.1494

**Published:** 2024-02-29

**Authors:** Ruth A Purcell, L Carissa Aurelia, Robyn Esterbauer, Lilith F Allen, Katherine A Bond, Deborah A Williamson, Janine M Trevillyan, Jason A Trubiano, Jennifer J Juno, Adam K Wheatley, Miles P Davenport, Thi HO Nguyen, Katherine Kedzierska, Stephen J Kent, Kevin John Selva, Amy W Chung

**Affiliations:** ^1^ Department of Microbiology and Immunology The Peter Doherty Institute for Infection and Immunity, University of Melbourne Melbourne VIC Australia; ^2^ Victorian Infectious Diseases Reference Laboratory (VIDRL) The Peter Doherty Institute for Infection and Immunity Melbourne VIC Australia; ^3^ Walter and Eliza Hall Institute of Medical Research Parkville VIC Australia; ^4^ Department of Infectious Diseases The Peter Doherty Institute for Infection and Immunity, University of Melbourne Melbourne VIC Australia; ^5^ Centre for Antibiotic Allergy and Research, Department of Infectious Diseases Austin Health Heidelberg VIC Australia; ^6^ Department of Medicine University of Melbourne Parkville VIC Australia; ^7^ Department of Infectious Diseases Peter MacCallum Cancer Centre Melbourne VIC Australia; ^8^ National Centre for Infections in Cancer Peter MacCallum Cancer Centre Melbourne VIC Australia; ^9^ Kirby Institute University of New South Wales Kensington NSW Australia; ^10^ Melbourne Sexual Health Centre and Department of Infectious Diseases, Alfred Health, Central Clinical School Monash University Melbourne VIC Australia

**Keywords:** allotype, anti‐immunoglobulin, IgG, polymorphisms, reproducibility, serology

## Abstract

**Objectives:**

Amino acid variations across more than 30 immunoglobulin (Ig) allotypes may introduce structural changes that influence recognition by anti‐Ig detection reagents, consequently confounding interpretation of antibody responses, particularly in genetically diverse cohorts. Here, we assessed a panel of commercial monoclonal anti‐IgG1 clones for capacity to universally recognise two dominant IgG1 haplotypes (G1m‐1,3 and G1m1,17).

**Methods:**

Four commercial monoclonal anti‐human IgG1 clones were assessed via ELISAs and multiplex bead‐based assays for their ability to bind G1m‐1,3 and G1m1,17 IgG1 variants. Detection antibodies were validated against monoclonal IgG1 allotype standards and tested for capacity to recognise antigen‐specific plasma IgG1 from G1m‐1,3 and G1m1,17 homozygous and heterozygous SARS‐CoV‐2 BNT162b2 vaccinated (*n* = 28) and COVID‐19 convalescent (*n* = 44) individuals. An Fc‐specific *pan*‐IgG detection antibody corroborated differences between hinge‐ and Fc‐specific anti‐IgG1 responses.

**Results:**

Hinge‐specific anti‐IgG1 clone 4E3 preferentially bound G1m1,17 compared to G1m‐1,3 IgG1. Consequently, SARS‐CoV‐2 Spike‐specific IgG1 levels detected in G1m1,17/G1m1,17 BNT162b2 vaccinees appeared 9‐ to 17‐fold higher than in G1m‐1,3/G1m‐1,3 vaccinees. Fc‐specific IgG1 and *pan*‐IgG detection antibodies equivalently bound G1m‐1,3 and G1m1,17 IgG1 variants, and detected comparable Spike‐specific IgG1 levels between haplotypes. IgG1 responses against other human coronaviruses and influenza were similarly poorly detected by 4E3 anti‐IgG1 in G1m‐1,3/G1m‐1,3 subjects.

**Conclusion:**

Anti‐IgG1 clone 4E3 confounds assessment of antibody responses in clinical cohorts owing to bias towards detection of G1m1,17 IgG1 variants. Validation of anti‐Ig clones should include evaluation of binding to relevant antibody variants, particularly as the role of immunogenetics upon humoral immunity is increasingly explored in diverse populations.

## Graphical abstract

Immunoglobulins comprise two fragment antigen‐binding (Fab) regions and a fragment crystallisable (Fc) region connected by a flexible hinge. Common IgG1 allotypes and corresponding polymorphisms are depicted. Here we show that some commercially available anti‐IgG1 detectors bind to IgG1 allotypes differently. This can confound assessment of antibody responses in genetically diverse cohorts.

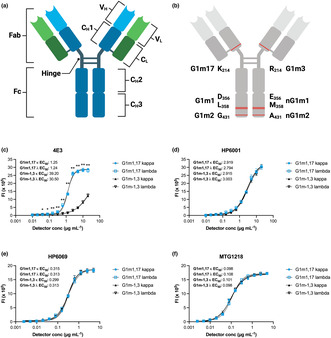

## Introduction

Reliable antibody‐based detection reagents are fundamental to biomedical research, and antibody detectors targeted against immunoglobulin (Ig) molecules are of particular importance in immunological and clinical research. However, insufficient validation or inappropriate use of commercial antibody‐based tools continues to contribute substantially to the reproducibility crisis pervasive within biomedical research.^1^, ^2^ As such, it is critical that investigators are aware of the importance of thoroughly validating anti‐IgG reagents for use in desired applications.

**Figure 1:**
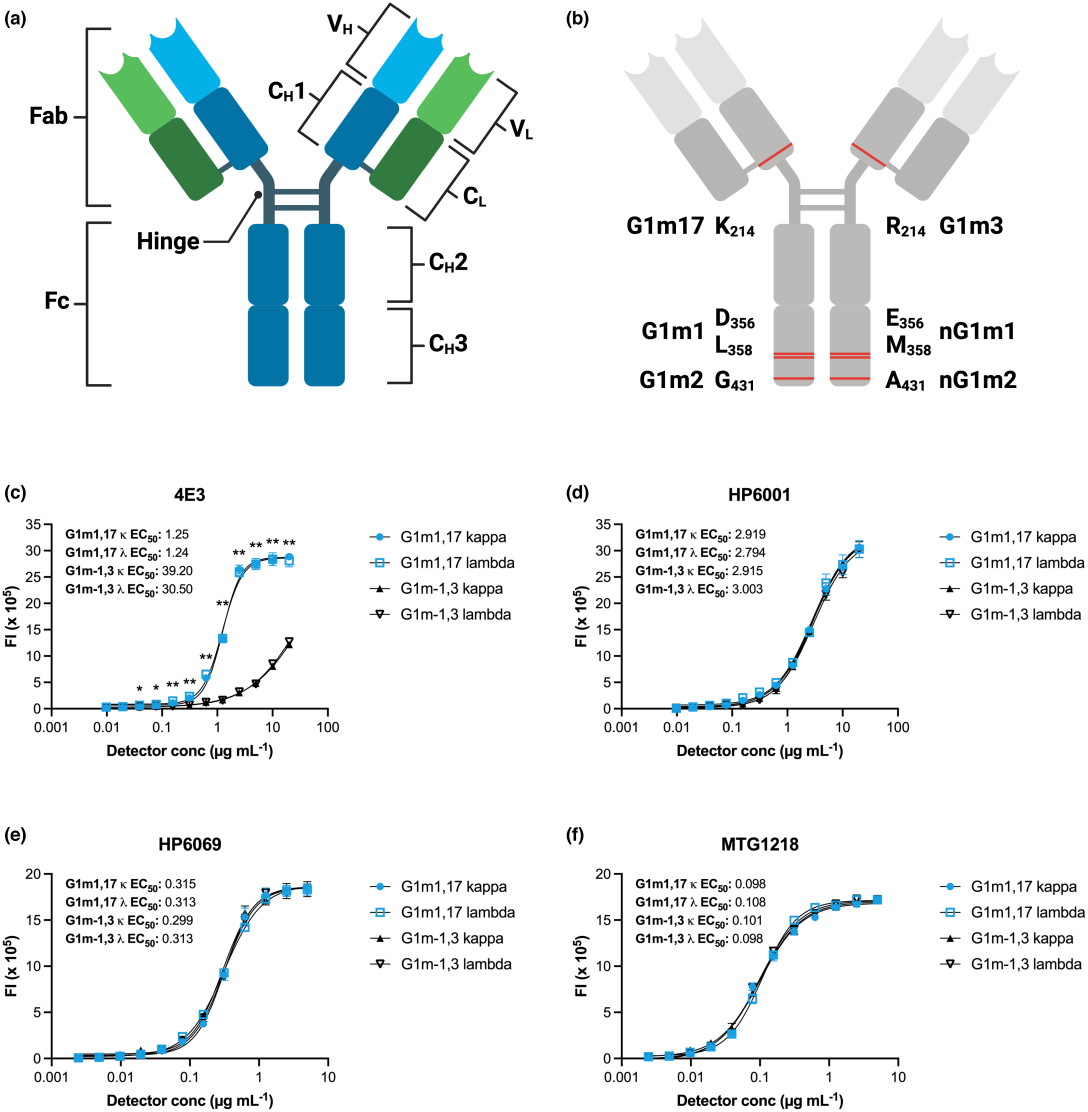
**(a)** Structure of an IgG1 antibody. Immunoglobulins comprise two fragment antigen‐binding (Fab) regions and a fragment crystallisable (Fc) region connected by a flexible hinge. Two constant heavy (C_H_) and two constant light (C_L_) chains are connected via disulphide bridges, which, along with two variable heavy (V_H_) and two variable light (V_L_) chains, form an ~150 kDa molecule. The C_H_ chain comprises three structural regions (C_H_1‐C_H_3) as well as the flexible hinge. **(b)** IgG polymorphisms giving rise to allotypes are located within the C_H_1 and C_H_3 regions of IgG1. EC_50_s of **(c)** 4E3, **(d)** HP6001, **(e)** HP6069 and **(f)** MTG1218 anti‐IgG1 clones for kappa and lambda variants of G1m‐1,3 and G1m1,17 allotype mAbs. Measurements were performed in triplicate and mean values ± SEM are indicated. Curves were fitted using a four‐parameter nonlinear regression. Mann–Whitney *U*‐tests were performed between responses against G1m1,17 (kappa and lambda) and G1m‐1,3 (kappa and lambda) allotype mAb standards within each concentration of 4E3 anti‐IgG1 detection reagent. *P* < 0.01 (**); *P* < 0.05 (*).

Anti‐Ig antibodies are raised against the constant heavy chain (C_H_) of an antibody molecule (Figure 1a) which, in theory, should render these reagents specific to distinct antibody isotypes (IgA, IgD, IgE, IgG and IgM) or subclasses (IgA1‐2 and IgG1‐4). However, despite its name, the antibody constant region contains a diverse array of single nucleotide polymorphisms (SNPs) that introduce considerable variation at the amino acid level. This sequence diversity has been most comprehensively characterised for IgG and IgA, with IgG3, followed by IgG1, demonstrating the most extensive variation.^3^

These Ig polymorphisms were initially termed ‘allotypes’ following identification in 1956 via rabbit anti‐sera produced by animals inoculated with serum from an immunogenetically distinct counterpart.^4^ Almost three decades later, monoclonal anti‐allotype antibody detectors were developed.^5^ Given the immunogenic nature of allotypes, it is not surprising that these alleles underlie variable interactions between immunoglobulins and their respective anti‐isotype detection reagents. Although individual allotypic markers are typically defined by only one or two amino acid substitutions, these substitutions may alter protein–protein interactions by mutating the epitope recognised by the detection antibody, interfering with binding via steric hindrance, or by inducing conformational changes at the binding site of the detection antibody, even if this epitope is distant from the position of the substituted amino acids.^6^ Nevertheless, despite the initial identification of allotypes via serological methods, thorough validation of binding by antibody detection reagents to different Ig allotypes is not routinely performed.

IgG1 is the most abundant immunoglobulin in humans, constituting approximately 65% of IgG.^7^, ^8^ IgG1 is important for protection against infectious agents^3^, ^7^, ^9^, ^10^ and cancer,^11^, ^12^ while also implicated in autoimmune diseases.^13^, ^14^ Four IgG1 allotypic markers (G1m) have been defined: G1m1, G1m2, G1m3 and G1m17 (Figure 1b). G1m3 and G1m17 are antithetical markers, while the alternate alleles for G1m1 and G1m2 allotypes are denoted as the absence of the marker (nG1m1/G1m‐1 and nG1m2, respectively). As immunoglobulin allotypes are inherited in a Mendelian fashion, IgG1 haplotypes tend to cluster ethnically and geographically. For example, the G1m‐1,3 haplotype is dominant in individuals of European descent, while the G1m1,17 haplotype is more prevalent, reaching frequencies over 80%, within African and Asian populations as well as American and Australian First Nations peoples.^3^, ^15^, ^16^

Here, we demonstrate that certain commercial anti‐IgG1 antibodies display variable binding to G1m‐1,3 and G1m1,17 IgG1 variants. We show that a detection antibody targeting the IgG1 hinge region preferentially binds G1m1,17 over G1m‐1,3 variants, while IgG1 and *pan*‐IgG clones raised against the Fc portion bind G1m‐1,3 and G1m1,17 IgG1 equivalently. These data emphasise the importance of thoroughly validating the suitability of antibody detection reagents for the characterisation of humoral responses, particularly in small clinical cohorts comprising genetically diverse individuals.

## Results

### Anti‐human IgG1 detector 4E3 displays limited binding to the G1m‐1,3 IgG1 allotype variant

Given the known antigenic nature of IgG1 allotype variants, we sought to determine whether these amino acid modifications influence binding interactions with anti‐human IgG1 antibody detection reagents. We characterised the binding of the anti‐IgG1 clones 4E3, HP6001, HP6069 and MTG1218 to kappa and lambda light chain variants of G1m‐1,3 and G1m1,17 IgG1 allotype mAb standards. The hinge‐specific anti‐IgG1 clone 4E3 demonstrated preferential binding to the G1m1,17 haplotype (κ, EC_50_ = 1.25; λ, EC_50_ = 1.24) but limited binding to G1m‐1,3 (κ, EC_50_ = 39.20; λ, EC_50_ = 30.50; Figure 1c). Binding to G1m1,17 was significantly increased (*P* = 0.026–*P* = 0.0022) within the detector dilution range of 20–0.04 μg mL^−1^. The Fc‐specific clones HP6001 and HP6069, as well as MTG1218, bound G1m‐1,3 and G1m1,17 allotype mAb standards equivalently (Figure 1d–f). Notably, in the context of a single G1m haplotype, binding to kappa and lambda variants was comparable (Figure 1c–f).

### Anti‐IgG1 clone influences antigen‐specific IgG1 levels detected in patient samples

Following the observation that the 4E3 anti‐IgG1 clone, but not the MTG1218, HP6001 and HP6069 clones, differentially bind G1m‐1,3 and G1m1,17 allotype mAb standards (Figure 1c–f), we next aimed to determine whether the detector clone influenced quantification of antigen‐specific IgG1 levels in a small clinical cohort. Using a custom Luminex bead‐based multiplex assay, IgG1 levels against SARS‐CoV‐2 and influenza were assessed in homozygous G1m‐1,3/G1m‐1,3 and G1m1,17/G1m1,17 BNT162b2 vaccinees using each of the four anti‐IgG1 detection reagents and compared between haplotypes.

**Figure 2:**
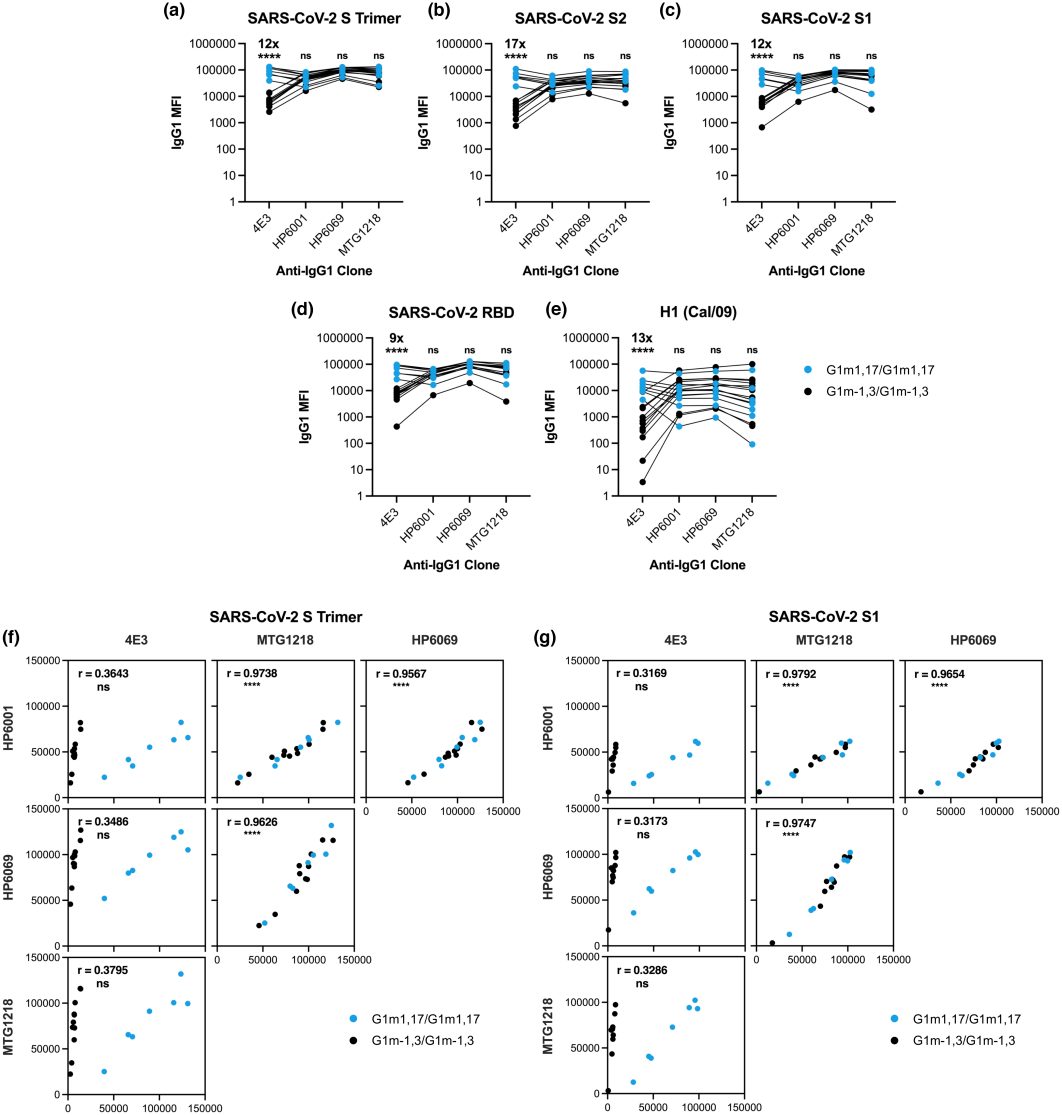
Plasma IgG1 levels of two‐dose BNT162b2 vaccinees against **(a)** SARS‐CoV‐2 S Trimer, **(b)** SARS‐CoV‐2 S2, **(c)** SARS‐CoV‐2 S1, **(d)** SARS‐CoV‐2 RBD and **(e)** H1 (Cal/09) measured with 4E3, HP6001, HP6069, or MTG1218 anti‐IgG1 clones. Mann–Whitney *U*‐tests were performed between G1m1,17/G1m1,17 and G1m‐1,3/G1m‐1,3 individuals within each anti‐IgG1 clone. Fold changes indicate relative increases in antigen‐specific IgG1 detected in G1m1,17/G1m1,7 compared to G1m‐1,3/G1m‐1,3 vaccinees. Correlations between anti‐SARS‐CoV‐2 IgG1 levels measured with each of 4E3, HP6001, HP6069 and MTG1218 anti‐IgG1 clones for representative SARS‐CoV‐2 antigens **(f)** S Trimer, **(g)** S1. The Shapiro–Wilk test confirmed normally of data distribution and the Pearson correlation coefficient (*r*) was computed. Black dots represent G1m‐1,3/G1m‐1,3 individuals (*n* = 11); Blue dots represent G1m1,17/G1m1,17 individuals (*n* = 7). *P* < 0.0001 (****); non‐significant (ns).

As expected, following the observation of preferential binding by hinge‐specific 4E3 to G1m1,17 allotype mAb standards compared to G1m‐1,3 mAbs (Figure 1c), the anti‐IgG1 clone 4E3 demonstrated enhanced detection of anti‐SARS‐CoV‐2 IgG1 in G1m1,17/G1m1,17 vaccinees (Figure 2a–d). Measurements obtained using 4E3 suggested that G1m1,17/G1m1,17 individuals mounted 9‐ to 17‐fold higher IgG1 responses against a range of SARS‐CoV‐2 antigens (S Trimer, S2, S1 and RBD) compared to G1m‐1,3/G1m‐1,3 individuals (*P* < 0.0001; Figure 2a–d; Supplementary figure 1). However, when antigen‐specific IgG1 levels were quantified using HP6001, HP6069, or MTG1218 anti‐IgG1 clones, no significant differences were observed between the IgG1 responses mounted by G1m‐1,3/G1m‐1,3 and G1m1,17/G1m1,17 vaccinees (Figure 2a–d). Notably, IgG1 levels against the unrelated antigen H1 (Cal/09) followed the same trend of detector‐driven variation whereby IgG1 responses quantified using the 4E3 clone were 13‐fold higher for G1m1,17/G1m1,17 than G1m‐1,3/G1m‐1,3 individuals (*P* < 0.0001) but not significant upon quantification with other anti‐IgG1 clones (Figure 2e). These influenza‐specific data are particularly striking given that this small SARS‐CoV‐2 vaccinated cohort would presumably have a highly heterogenous influenza infection and vaccination history.

Correlation of IgG1 levels in BNT162b2 vaccinated individuals measured using each of the 4E3, HP6001, HP6069 or MTG1218 anti‐IgG1 clones, indicated similar binding behaviour of HP6001, HP6069 and MTG1218 (*r* = 0.96–0.98), while 4E3 showed limited correlation with any other anti‐IgG1 clone (*r* = 0.32–0.38; Figure 2f, g). Indeed, correlation of IgG1 responses measured with 4E3 and each of HP6001, HP6069 or MTG1218 resulted in two distinct clusters (Figure 2f, g). One cluster corresponded to G1m1,17/G1m1,17 individuals for whom reasonable correlation between 4E3 and other anti‐IgG1 clones was observed. The alternate cluster corresponded to G1m‐1,3/G1m‐1,3 individuals for whom no correlation between 4E3 and other clones was evident. These correlations reinforce the conclusion that 4E3 does readily not bind the G1m‐1,3 IgG1 variant, as suggested by the EC_50_s calculated for this detector (Figure 1c), as well as comparisons between IgG1 responses measured using 4E3 and alternative anti‐IgG1 clones (Figure 2a–e).

### Anti‐IgG1 clone 4E3 may confound assessment of vaccination and infection induced antibodies from clinical cohorts

The potential for hinge‐specific anti‐IgG1 detector clone 4E3 to artificially bias antibody levels measured for clinical cohorts was next demonstrated in BNT162b2 vaccinated and COVID‐19 convalescent cohorts that included G1m‐1,3/G1m1,17 heterozygous individuals. Given that responses measured with the Fc‐specific clones HP6001 and HP6069, as well as MTG1218, strongly correlated, only 4E3 and HP6001 were used for further comparison.

**Figure 3:**
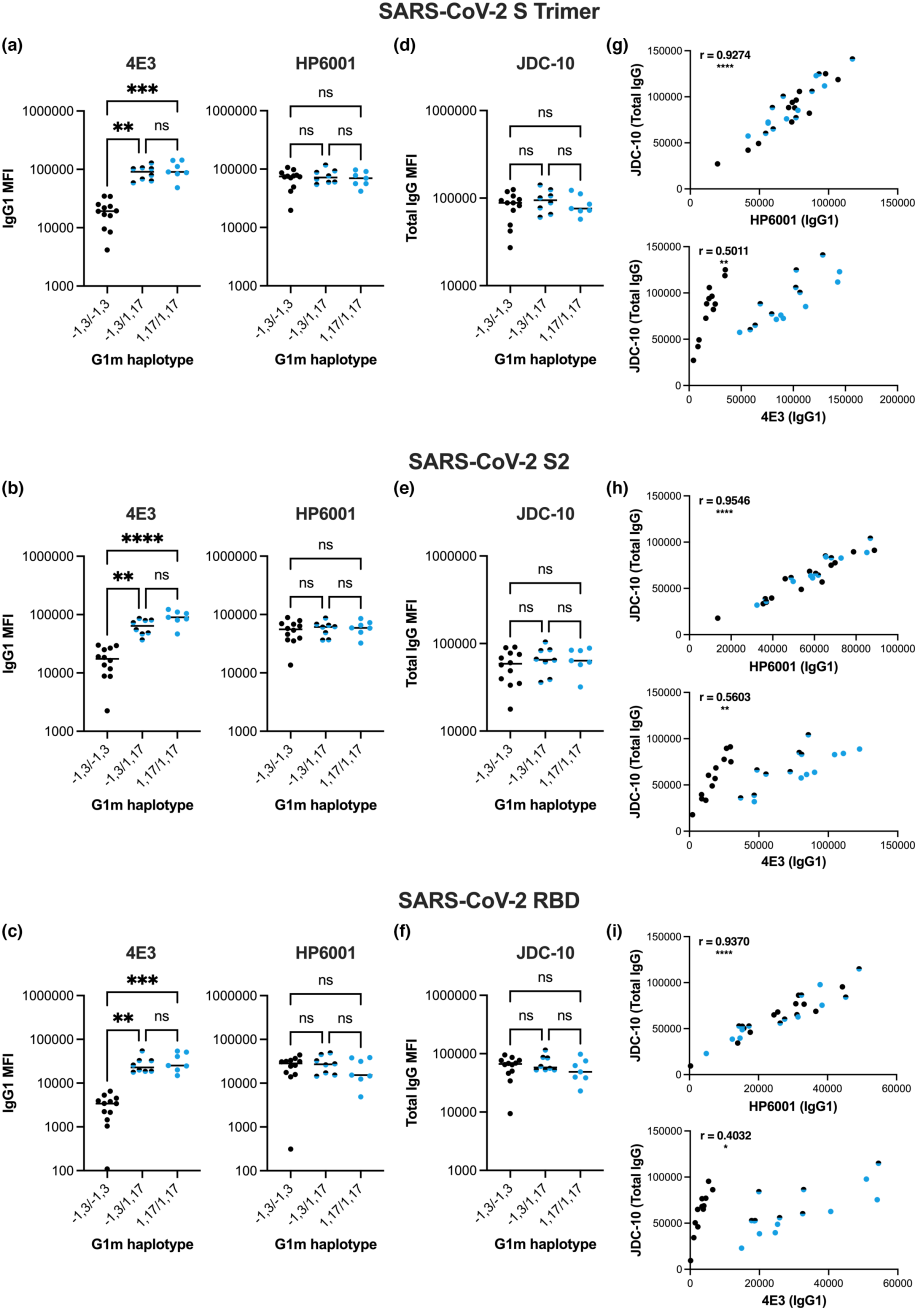
Plasma IgG1 levels of BNT162b2 vaccinated individuals against SARS‐CoV‐2 **(a)** S Trimer, **(b)** S2, **(c)** RBD measured with 4E3 or HP6001 anti‐IgG1 clones. Total plasma IgG levels of BNT162b2 vaccinated individuals against SARS‐CoV‐2 **(d)** S Trimer, **(e)** S2, **(f)** RBD measured with *pan*‐IgG detection antibody JDC‐10. Median values are represented by horizontal black lines. The Kruskal–Wallis test followed by Dunn's multiple comparisons test were performed between G1m‐1,3/G1m‐1,3, G1m‐1,3/G1m1,17 and G1m1,17/G1m1,17 individuals within each clone. Correlations between anti‐SARS‐CoV‐2 IgG1 and total IgG levels measured with 4E3 (anti‐IgG1), HP6001 (anti‐IgG1) or JDC‐10 (anti‐IgG) clones for SARS‐CoV‐2 **(g)** S Trimer, **(h)** S2 and **(i)** RBD. The Shapiro–Wilk test confirmed normally of data distribution and the Pearson correlation coefficient (*r*) was computed. Black dots represent G1m‐1,3/G1m‐1,3 homozygous individuals (*n* = 12); Black and blue dots represent G1m‐1,3/G1m1,17 heterozygous individuals (*n* = 8); Blue dots represent G1m1,17/G1m1,17 homozygous individuals (*n* = 7). *P* < 0.0001 (****); *P* < 0.001 (***); *P* < 0.01 (**); *P* < 0.05 (*); non‐significant (ns).

**Figure 4:**
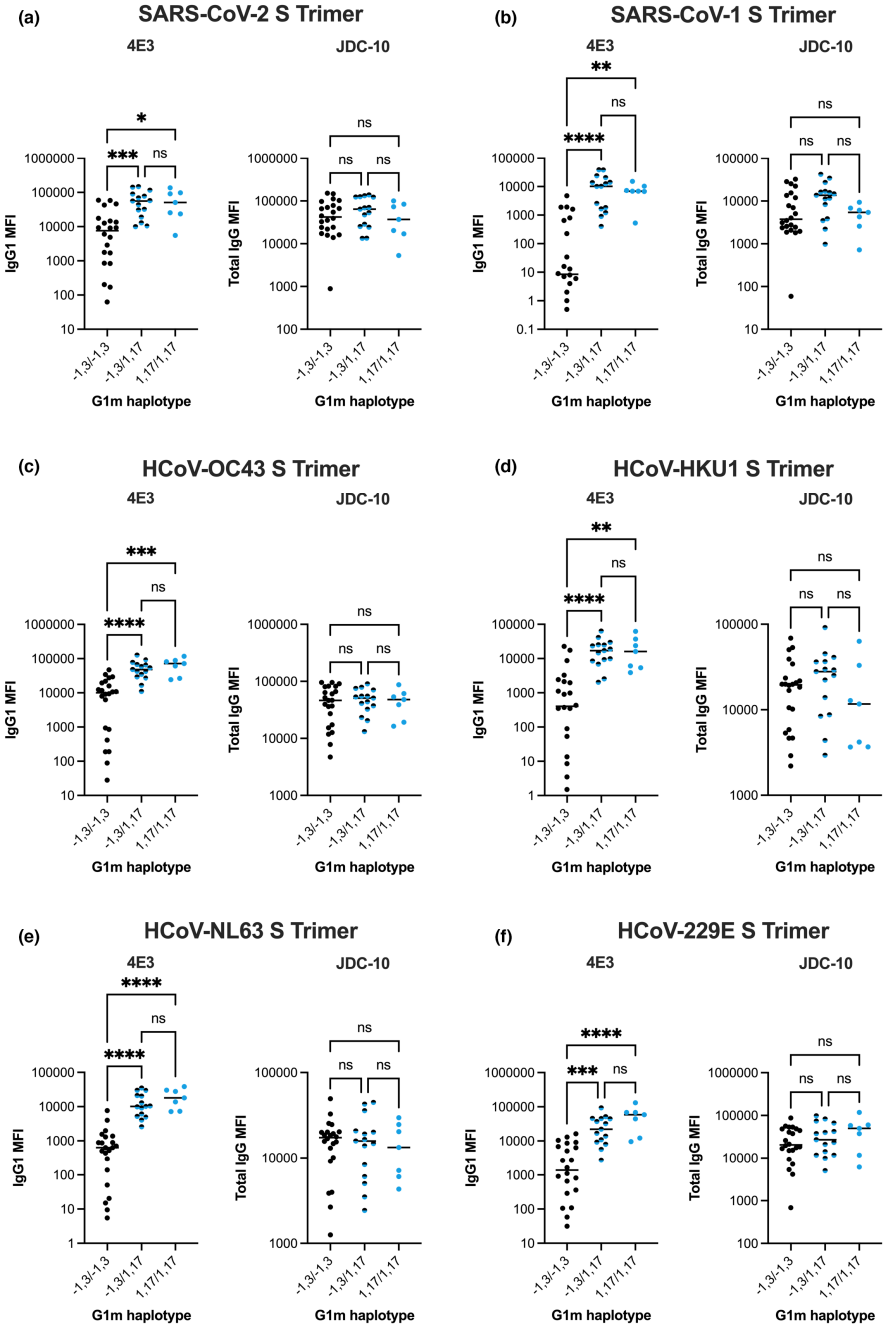
Plasma IgG1 and total IgG levels of mild–moderate COVID‐19 convalescent individuals against **(a)** SARS‐CoV‐2 S Trimer, **(b)** SARS‐CoV‐1 S Trimer, **(c)** HCoV‐OC43 S Trimer, **(d)** HCoV‐HKU1 S Trimer, **(e)** HCoV‐NL63 S Trimer, **(f)** HCoV‐229E S Trimer measured with 4E3 anti‐IgG1 detection antibody or JDC‐10 *pan*‐IgG detection antibody. Black dots represent G1m‐1,3/G1m‐1,3 homozygous individuals (*n* = 22); Black and blue dots represent G1m‐1,3/G1m1,17 heterozygous individuals (*n* = 15); Blue dots represent G1m1,17/G1m1,17 homozygous individuals (*n* = 7). Median values are represented by horizontal black lines. Kruskal–Wallis tests followed by Dunn's multiple comparisons tests were performed between G1m‐1,3/G1m‐1,3, G1m‐1,3/G1m1,17 and G1m1,17/G1m1,17 individuals within each detection reagent. *P* < 0.0001 (****); *P* < 0.001 (***); *P* < 0.01 (**); *P* < 0.05 (*); non‐significant (ns).

In line with previously observed trends (Figure 2a–e), IgG1 levels measured with 4E3, but not HP6001, were significantly higher for G1m1,17/G1m1,17 and G1m‐1,3/G1m1,17 than G1m‐1,3/G1m‐1,3 individuals across all antigens tested (*P* < 0.01 to *P* < 0.0001; Figure 3a–c). This result was further corroborated by the lack of G1m haplotype‐associated differences detected with the Fc‐specific anti‐human *pan*‐IgG clone JDC‐10 (Figure 3d–f). Given that IgG1 comprises approximately 90% total IgG post second dose BNT162b2 vaccination,^17^ the majority of IgG detected with the JDC‐10 clone is presumably IgG1. As such, it would be expected that IgG1 subclass and total IgG responses should strongly correlate. Robust correlations were observed between IgG1 and total IgG responses when IgG1 was quantified using HP6001, but not 4E3 (Figure 3g–i). Accordingly, when anti‐IgG clone JDC‐10 was used, no differences in total IgG levels were detected between G1m‐1,3/G1m‐1,3 and G1m‐1,3/G1m1,17 or G1m1,17/G1m1,17 individuals. Finally, in an independent cohort of mild–moderate COVID‐19 convalescents, the same trend of increased IgG1 (detected with 4E3), but not total IgG (detected with JDC‐10), in G1m‐1,3/G1m1,17 and G1m1,17/G1m1,17 individuals was observed across a panel of six pandemic and seasonal coronavirus spike trimers (Figure 4a–f), as well as SARS‐CoV‐2 and influenza antigens (Supplementary figure 2).

## Discussion

Antibody detection reagents frequently contribute to irreproducible studies within biomedical research.^1^, ^2^ This is typically a ramification of antibody cross‐reactivity or inappropriate use of antibody reagents in applications for which they have not been validated. Alternatively, as demonstrated here in the case of IgG allotypes, altered binding may occur when SNPs interfere with the capacity of detector reagents to recognise variants of the canonical protein isoform.

We demonstrate that the G1m1,17 IgG1 variant is preferentially bound by the hinge‐specific anti‐IgG1 detection antibody clone 4E3. Notably, the G1m3/G1m17 marker likely drives the IgG1 haplotype‐associated differential binding by 4E3. In contrast to Fc‐specific HP6001 and HP6069 anti‐IgG1 clones (the epitope of MTG1218 remains proprietary) as well as Fc‐specific *pan*‐IgG clone JDC‐10, 4E3 binds the hinge region. The G1m3/17 marker is located distally along the C_H_1 region at position 214, close to the IgG1 hinge (Figure 1b). The lysine (G1m17) to arginine (G1m3) substitution introduces a guanidine head group in place of the amino head group. Although lysine and arginine are both basic amino acids with similar chemical properties, the additional side chain present in arginine may impair binding of G1m‐1,3 IgG1 by hinge‐specific 4E3. However, the possibility of a singular or combinatorial influence of the G1m1/nG1m1 allotype upon detection of IgG1 by 4E3 cannot be excluded. Furthermore, the influence of the G1m2/nG1m2 allotype upon anti‐IgG binding remains to be determined.

Assessment of Ig detection reagent binding for universal allotype compatibility is essential in immunogenetics‐focused investigations. Previous studies have identified IgG haplotype‐associated differences in total and antigen‐specific Ig titres.^18^, ^19^, ^20^, ^21^, ^22^, ^23^, ^24^ However, a compelling mechanism for this phenomenon has yet to be elucidated. We demonstrate that when assay readouts directly depend upon binding by anti‐Ig detection reagents (e.g. ELISA, radioimmunoassay, radial immunodiffusion and variations thereof), equivalent detection antibody binding to all represented haplotypes should be confirmed prior to assessing immunogenetic influences upon antibody responses.

Promisingly, studies of allotype‐associated immune response variations have not been restricted to quantitative serology‐based assays susceptible to the confounding influence of inadequately validated detection antibodies. Enhanced disease protection^23^, ^25^ and functional immune responses^26^ have also been associated with various IgG haplotypes, implying that associations observed between IgG allotypes and altered antibody levels cannot be discounted, but should be confirmed with thoroughly validated detection reagents. As such, this topic warrants meticulous further investigation.

Awareness of the possible confounding influence of allotypes upon anti‐Ig detection antibody binding is of particular importance when assessing humoral responses in rare and unique clinical cohorts which typically rely on small sample sizes, especially when genetically diverse participants are recruited. In the likely instance that Ig allotypes are not equally represented across all study groups, inadequate anti‐Ig detection reagent validation may result in antibody responses for impacted experimental groups being artificially inflated if the selected detection antibody discriminates between allotypes. Critically, as Ig variants are increasingly described in populations historically underrepresented in the biomedical literature^27^ and as interest in the role of immunogenetics upon personalised vaccine responses heightens,^3^ the potential for host genetic variation to introduce experimental artefacts should be afforded greater consideration. Accordingly, investigators should thoroughly assess for the absence of any possible confounding interactions between novel alleles and antibody detection reagents. Such validation is essential for further work suggested by the present study as the influence of IgG1 allotypes upon SARS‐CoV‐2 antibody responses remains to be fully characterised in cohorts with more extensive coverage of homozygous and heterozygous G1m allelic combinations.

Here we have demonstrated the influence of G1m‐1,3 and G1m1,17 haplotypes upon binding of monoclonal anti‐IgG1 detection reagents to human IgG1. Although we have only characterised four anti‐IgG1 clones which cannot fully represent the diversity of commercially available anti‐IgG detection reagents, anti‐IgG1 clone 4E3 is extensively cited in the biomedical literature, particularly in clinical cohort studies.^28^, ^29^, ^30^ Furthermore, investigators should be aware that the phenomenon of allotype‐associated detection antibody incompatibility likely exists between variants of other Ig isotypes and subclasses and their respective detection reagents. Such ‘serological blind spots’ have been observed for IgG3 allotypes,^31^ consistent with the highly polymorphic nature of this subclass,^3^ but have also been observed within the more conserved IgG4 subclass.^31^ Nevertheless, other investigators assessing this phenomenon have not detected inconsistencies in binding to different allotypes within alternative panels of detection reagents, observing instead only cross‐reactivity between IgG subclasses.^32^

In conclusion, we highlight the critical importance of thoroughly validating commercial antibody detection reagents used for serological characterisation of clinical cohorts, particularly those that include immunogenetically diverse participants.

## Methods

### Monoclonal IgG1 allotype standards and anti‐IgG antibody detection reagents

Four monoclonal IgG1 allotype antibodies (allotype mAb standards) were used as target antigens to quantify the half maximal effective concentration (EC_50_) of a panel of commercial monoclonal mouse anti‐human IgG1 detection reagents. Both kappa and lambda light chain variants of the G1m‐1,3 and G1m1,17 allotype mAb standards were used to confirm that the sequence variations between kappa and lambda light chains did not influence binding of anti‐IgG1 detectors. All four allotype mAb standards were recombinant antibodies with specificity for green fluorescent protein. The details of the four allotype mAb standards are tabulated in Supplementary table 1.

Four monoclonal anti‐human IgG1 antibody detection reagents (clones 4E3, HP6001, HP6069 and MTG1218) were tested against the four allotype mAb standards and used to assess antibody levels in patient plasma. One monoclonal anti‐human *pan*‐IgG antibody detection reagent (clone JDC‐10) was used to assess antibody levels in patient plasma. The details of the five anti‐IgG antibody detection reagents are tabulated in Supplementary table 2.

### Study participants and sample collection

SARS‐CoV‐2 vaccinee plasma and granulocyte samples were collected from individuals 12–42 days following second dose BNT162b2 (Comirnaty; Pfizer‐BioNTech) vaccination (Total *n* = 28: G1m‐1,3/G1m‐1,3 (*n* = 13); G1m‐1,3/G1m1,17 (*n* = 8); G1m1,17/G1m1,17 (*n* = 7)), as previously described.^33^ Vaccinee cohort demographics and plasma sample details are described in Supplementary table 3. Mild–moderate COVID‐19 convalescent plasma and granulocyte samples were collected from individuals 8–58 days following positive SARS‐CoV‐2 test (Total *n* = 44: G1m‐1,3/G1m‐1,3 (*n* = 22); G1m‐1,3/G1m1,17 (*n* = 15); G1m1,17/G1m1,17 (*n* = 7)), as previously described.^34^ Convalescent cohort demographics and plasma sample details are described in Supplementary table 4. Whole blood was collected into sodium heparin anticoagulant coated vacutainers and subjected to Ficoll‐Paque (Cytiva, Uppsala, Sweden; 17144002) separation. Plasma and granulocytes were collected and stored at −80 °C. Study protocols were approved by the University of Melbourne Human Research Ethics Committee (#2056689, #21560, #21626 and #13344), Austin Health (#HREC/73256/Austin‐2021) and Melbourne Health (HREC/68355/MH‐2020).

### Enzyme‐linked immunosorbent assay (ELISA)

Nunc MaxiSorp flat‐bottom 96‐well plates (Thermo Fisher Scientific, Scoresby, VIC, Australia; 44‐2404) were coated overnight at 4 °C with 100 μg (for characterisation of HP6069 and MTG1218) or 200 μg (for characterisation of 4E3 and HP6001) of respective IgG1 allotype mAb standard (G1m‐1,3 kappa, Clone AbD18705_hIgG1 (Bio‐Rad, Gladesville, NSW, Australia; HCA192)); G1m‐1,3 lambda, Clone AbD00264_hIgG1 (Bio‐Rad, HCA049); G1m1,17 kappa, Clone AbD18705il (Bio‐Rad, HCA319); or G1m1,17 lambda, Clone AbD00264il (Bio‐Rad, HCA318), washed with PBST, then blocked with PBS containing 0.1% BSA (Sigma‐Aldrich, St. Louis, MO, USA; A7906‐100G) and 0.05% Tween 20 (Sigma‐Aldrich, P1379‐500ML), and again washed with PBST. Two‐fold dilutions of anti‐IgG1 detection antibodies (Mouse Anti‐Human IgG1 Hinge‐PE (4E3) (Southern Biotech, Birmingham, AL, USA; 9052‐09); Mouse Anti‐Human IgG1 Fc‐PE (HP6001; Southern Biotech, 9054‐09); HP6069 (Merck, Bayswater, VIC, Australia; 411543‐200UG); MTG1218 (MabTech, Cincinnati, OH, USA; 3851‐14‐250) were prepared in blocking solution (PBST containing 0.1% BSA), beginning at 5 μg mL^−1^ for MTG1218 and HP6069 and beginning at 20 μg mL^−1^ for HP6001 and 4E3. Anti‐human IgG1 antibody detection reagent dilutions were incubated with allotype mAb standards coated on the ELISA plate for 2 h at room temperature. ELISAs performed with biotinylated MTG1218 and HP6069 were washed five times with PBST then incubated for a further 2 h at room temperature with 1 μg mL^−1^ Streptavidin, R‐Phycoerythrin Conjugate (SAPE; Thermo Fisher Scientific, S866), followed by a final five washes with PBST. ELISAs performed with R‐Phycoerythrin (PE)‐conjugated 4E3 and HP6001 were directly read following a final five washes with PBST. Fluorescence intensity was read on a CLARIOstar *Plus* (BMG Labtech, Mornington, VIC, Australia) microplate reader with excitation wavelength of 488‐15 nm and emission of 576‐20 nm using the Enhanced Dynamic Range (EDR) function, selected based on the excitation (496 and 565 nm) and emission (578 nm) maxima of PE. Technical replicates were performed in triplicate.

### DNA extraction, polymerase chain reaction (PCR) and sequencing

G1m1/nG1m1 and G1m3/G1m17 typing of study participants was performed via PCR, as previously described,^35^, ^36^ with minor modifications. Genomic DNA was extracted from granulocytes using the QIAamp DNA Blood Mini Kit (Qiagen GmbH, Hilden, Germany; 51104) according to the manufacturer's instructions. Amplification of the human C_H_1 and C_H_3 domains of *IGHG1* was performed using the AccuPrime *Taq* DNA Polymerase, High Fidelity system (Thermo Fisher Scientific, 12346094) prepared with 150 ng DNA template, 3 μL 10× AccuPrime PCR buffer II, 5 units per μL enzyme and 0.25 μm each primer, brought to a total volume of 30 μL in nuclease‐free water. Initial denaturation was performed at 94 °C for 30 s followed by 35 cycles of denaturation at 94 °C for 25 s, annealing at 62 °C for 25 s and extension at 72 °C for 50 s, with a final extension at 72 °C for 7 min. Dual direction sequencing of PCR products was performed by the Australian Genome Research Facility (AGRF, Melbourne, VIC, Australia). Geneious Prime version 2023.2.1 was used for sequence analysis and genotypes were manually called.

### Luminex bead‐based multiplex assay

To characterise antigen‐specific plasma antibody responses, we utilised a panel of severe acute respiratory syndrome coronavirus 2 (SARS‐CoV‐2), SARS‐CoV‐1, seasonal human coronaviruses (HCoV‐OC43, HCoV‐HKU1, HCoV‐NL63, HCoV‐229E), and influenza antigen‐coupled beads in a custom Luminex multiplex array, validated as previously described.^33^, ^37^ Briefly, magnetic carboxylated beads (Bio‐Rad) were coupled with 100 μg antigen (SARS‐CoV‐2 Spike 1 (S1), S2, SARS‐CoV‐2 receptor binding domain (RBD), and haemagglutinin H1 (A/California/07/2009; H1 (Cal/09); Sino Biological, Beijing, China)) or 30 μg (SARS‐CoV‐2, HCoV‐OC43, HCoV‐HKU1, HCoV‐NL63 and HCoV‐229E S Trimers – gifts from Adam Wheatley) per 1.25 × 10^7^ beads. One thousand beads/bead region diluted in PBS containing 0.1% BSA (incubation buffer) were added to each well (in a total of 25 μL per well) in black, clear‐bottom 384‐well plates (Greiner Bio‐One, Kremsmünster, Austria; 781906), followed by addition of 25 μL per well of plasma diluted in PBS at a single concentration (1:100 for convalescent plasma and 1:800 for vaccinee plasma). Plates were incubated on a plate shaker overnight at 4 °C before wells were washed with PBS plus 0.05% Tween‐20 (PBST). PE‐conjugated mouse anti‐human IgG1 (4E3 (Southern Biotech, 9052‐09) or HP6001 (Southern Biotech, 9054‐09)), PE‐conjugated mouse anti‐human *pan*‐IgG (JDC‐10 (Southern Biotech, 9040‐09)), or biotinylated mouse anti‐human IgG1 (HP6069 (Merck, 411543‐200UG) or MTG1218 (MabTech, 3851‐14‐250)) were diluted to 1.3 μg mL^−1^ and added at 25 μL per well before incubation for 2 h at room temperature on a plate shaker. For biotinylated detectors, plates were followed with an additional PBST wash, and then incubated with 1 μg mL^−1^ SAPE (Thermo Fisher Scientific, S866) at room temperature for 2 h. Plates were then washed with PBST, and beads were resuspended in 50 μL of sheath fluid per well. The level of PE signal associated with each bead region in each well, reported as median fluorescence intensity (MFI), was determined by a FLEXMAP 3D Luminex instrument system (Luminex, Austin, TX, USA). Each sample was run in duplicate.

### Statistical analysis

Prism GraphPad version 10.1.0 (GraphPad Software, San Diego, CA, USA) was used to develop graphs and perform the statistical analyses described in the figure captions. Half maximal effective concentration (EC_50_) of each anti‐IgG1 detector was determined using a four‐parameter nonlinear regression model describing the relationship between agonist concentration and response.

## Author contributions

**Ruth A Purcell:** Conceptualization; formal analysis; investigation; methodology; writing – original draft; writing – review and editing. **L Carissa Aurelia:** Formal analysis; investigation; methodology. **Robyn Esterbauer:** Investigation. **Lilith F Allen:** Investigation. **Katherine A Bond:** Investigation. **Deborah A Williamson:** Investigation. **Janine M Trevillyan:** Investigation; writing – review and editing. **Jason A Trubiano:** Funding acquisition; investigation; writing – review and editing. **Jennifer J Juno:** Funding acquisition; investigation; project administration. **Adam K Wheatley:** Funding acquisition; investigation; resources. **Miles P Davenport:** Funding acquisition; investigation. **Thi HO Nguyen:** Funding acquisition; investigation; project administration; writing – review and editing. **Katherine Kedzierska:** Funding acquisition; investigation; project administration. **Stephen J Kent:** Funding acquisition; investigation; project administration; supervision; writing – review and editing. **Kevin John Selva:** Investigation; supervision; writing – review and editing. **Amy W Chung:** Conceptualization; formal analysis; funding acquisition; investigation; methodology; project administration; supervision; writing – review and editing.

## Conflict of interest

The authors declare no conflict of interest.

## Supporting information


Supporting Information

## Data Availability

The data that support the findings of this study are available from the corresponding author upon reasonable request.
