# Supplementary material for: Immunoglobulin G genetic variation can confound assessment of antibody levels via altered binding to detection reagents
Source: Clin Transl Immunology. 2024 Feb 29;13(3):e1494. doi: 10.1002/cti2.1494 (PMC10902689; doi:10.1002/cti2.1494)
Supplement: Supplementary file 1 — Supporting Information [file CTI2-13-e1494-s001.docx]

**
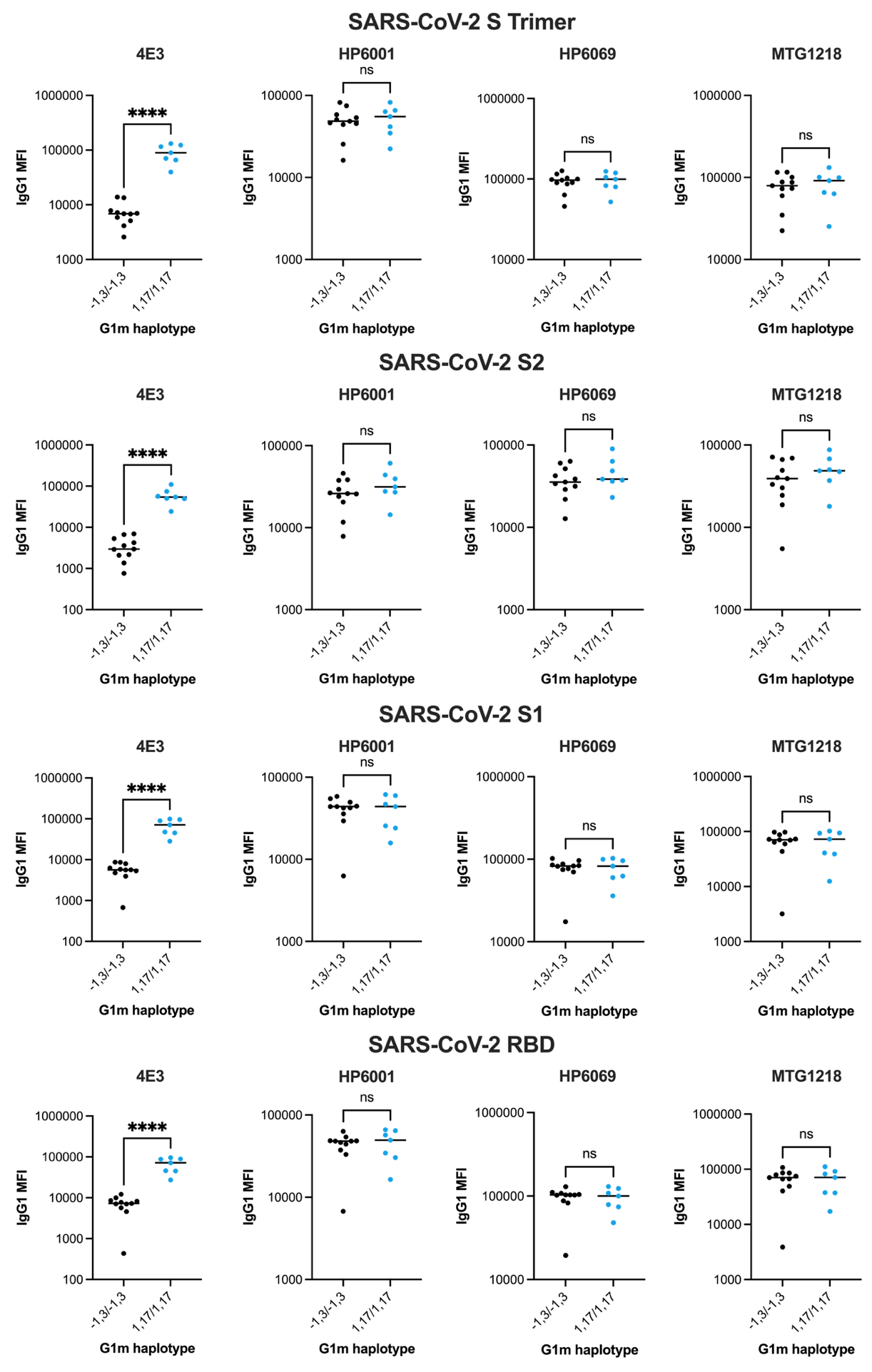
Supplementary Materials**

**Supplementary figure 1.** Plasma IgG1 levels of BNT162b2 vaccinated individuals against SARS-CoV-2 Trimer, S2, S1, and RBD measured with 4E3, HP6001, HP6069, or MTG1218 anti-IgG1 clones. Black dots represent G1m-1,3/G1m-1,3 individuals (*n* = 11); Blue dots represent G1m1,17/G1m1,17 individuals (*n* = 7). Median values are represented by horizontal black lines. Mann-Whitney *U*-tests were performed between G1m1,17 and G1m-1,3 homozygous individuals within each anti-IgG1 clone. *P* < 0.0001 (****); non-significant (ns).


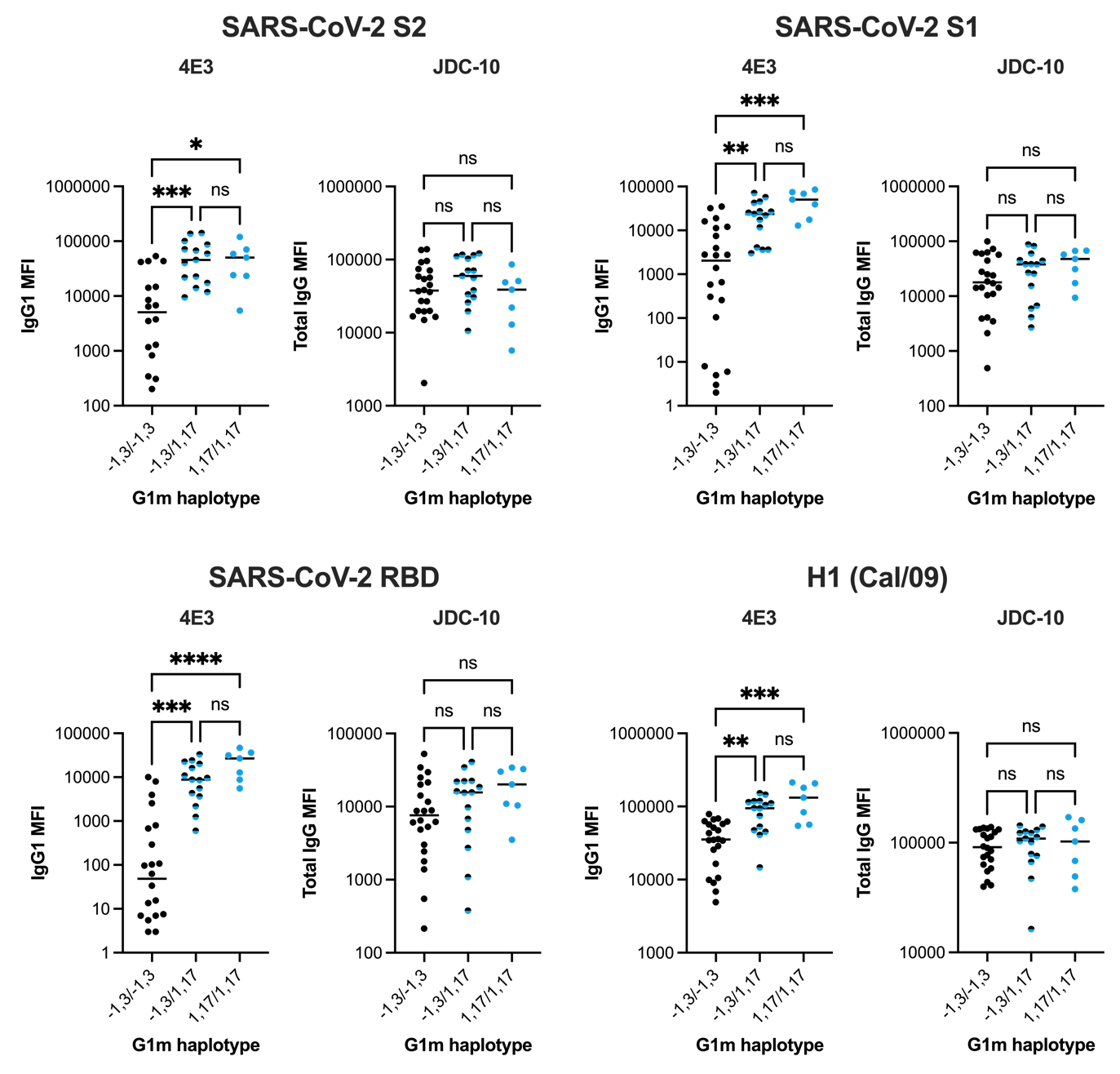


**Supplementary figure 2.** Plasma IgG1 and total IgG levels of mild-moderate COVID-19 convalescent individuals against SARS-CoV-2 S Trimer, SARS-CoV-2 S2, SARS-CoV-2 S1, SARS-CoV-2 RBD, and H1 (Cal/09) measured with 4E3 anti-IgG1 detection antibody or JDC-10 *pan*-IgG detection antibody. Black dots represent G1m-1,3/G1m-1,3 homozygous individuals (*n* = 22); Black and blue dots represent G1m-1,3/G1m1,17 heterozygous individuals (*n* = 15); Blue dots represent G1m1,17/G1m1,17 homozygous individuals (*n* = 7). Median values are represented by horizontal black lines. The Kruskal-Wallis test followed by Dunn’s multiple comparisons test were performed between G1m-1,3/G1m-1,3, G1m-1,3/G1m1,17, and G1m1,17/G1m1,17 individuals within each detection reagent. *P* < 0.0001 (****); *P* < 0.001 (***); *P* < 0.01 (**); *P* < 0.05 (*); non-significant (ns).

**Supplementary table 1. IgG1 allotype mAb standards**

| **IgG1 allotype mAb** | **G1m haplotype** | **Light chain** | **Clone** | **Specificity** | **Product number** |
| --- | --- | --- | --- | --- | --- |
| Recombinant Human IgG1 Kappa Allotype G1m3 | G1m-1,3 | Kappa | AbD18705_hIgG1 | Green Fluorescent Protein | HCA192 |
| Recombinant Human IgG1 Lambda Allotype G1m3 | G1m-1,3 | Lambda | AbD00264_hIgG1 | Green Fluorescent Protein | HCA049 |
| Recombinant Human IgG1 Kappa Allotype G1m17,1 | G1m1,17 | Kappa | AbD18705il | Green Fluorescent Protein | HCA319 |
| Recombinant Human IgG1 Lambda Allotype G1m17,1 | G1m1,17 | Lambda | AbD00264il | Green Fluorescent Protein | HCA318 |

**Supplementary table 2. Monoclonal anti-human IgG1 and *pan*-IgG antibody detectors**

| **Detector name** | **Clone** | **Specificity** | **Conjugate** | **Product number** |
| --- | --- | --- | --- | --- |
| Mouse Anti-Human IgG1 Hinge-PE (4E3) | 4E3 | IgG1 (Hinge) | Phycoerythrin (PE) | 9052-09 |
| Mouse Anti-Human IgG1 Fc-PE (HP6001) | HP6001 | IgG1 (Fc) | Phycoerythrin (PE) | 9054-09 |
| Mouse Anti-Human IgG₁, Fc Fragment Specific (HP6069) Biotin Conjugate | HP6069 | IgG1 (Fc) | Biotin | 411543-200UG |
| Anti-human IgG1 mAb (MTG1218), biotin | MTG1218 | IgG1 | Biotin | 3851-14-250 |
| Mouse Anti-Human IgG Fc-PE (JDC-10) | JDC-10 | IgG (Fc) | Phycoerythrin (PE) | 9040-09 |

**Supplementary table 3. BNT162b2 vaccinee cohort demographics and sample details**

| **Patient ID** | **G1m haplotype** | **Age** | **Sex** | **Sample timepoint (days post-vaccination)** |
| --- | --- | --- | --- | --- |
| VAX01 | G1m-1,3/G1m-1,3 | 31 | M | 30 |
| VAX02 | G1m-1,3/G1m1,17 | 49 | F | 30 |
| VAX03 | G1m-1,3/G1m1,17 | 37 | M | 30 |
| VAX04 | G1m-1,3/G1m1,17 | 30 | M | 42 |
| VAX05 | G1m-1,3/G1m-1,3 | 23 | F | 34 |
| VAX06 | G1m-1,3/G1m1,17 | 45 | F | 28 |
| VAX07 | G1m1,17/G1m1,17 | 46 | F | 34 |
| VAX08 | G1m-1,3/G1m-1,3 | 54 | F | 12 |
| VAX09 | G1m-1,3/G1m1,17 | 28 | F | 13 |
| VAX10 | G1m-1,3/G1m1,17 | 25 | F | 13 |
| VAX11 | G1m-1,3/G1m-1,3 | 31 | M | 14 |
| VAX12 | G1m-1,3/G1m-1,3 | 34 | F | 12 |
| VAX13 | G1m1,17/G1m1,17 | 29 | F | 13 |
| VAX14 | G1m-1,3/G1m-1,3 | 26 | F | 18 |
| VAX15 | G1m-1,3/G1m-1,3 | 25 | F | 14 |
| VAX16 | G1m-1,3/G1m1,17 | 35 | M | 13 |
| VAX17 | G1m-1,3/G1m-1,3 | 30 | M | 15 |
| VAX18 | G1m1,17/G1m1,17 | 57 | F | 14 |
| VAX19 | G1m1,17/G1m1,17 | 34 | M | 13 |
| VAX20 | G1m-1,3/G1m-1,3 | 57 | F | 13 |
| VAX21 | G1m-1,3/G1m1,17 | 65 | M | 14 |
| VAX22 | G1m-1,3/G1m-1,3 | 29 | M | 14 |
| VAX23 | G1m1,17/G1m1,17 | 49 | M | 14 |
| VAX24 | G1m-1,3/G1m-1,3 | 24 | F | 30 |
| VAX25 | G1m1,17/G1m1,17 | 38 | F | 30 |
| VAX26 | G1m1,17/G1m1,17 | 49 | F | 30 |
| VAX27 | G1m-1,3/G1m-1,3 | 56 | F | 30 |
| VAX28 | G1m-1,3/G1m-1,3 | 34 | F | 30 |

**Supplementary table 4. COVID-19 convalescent cohort demographics and sample details**

| **Patient ID** | **G1m haplotype** | **Disease severity** | **Age** | **Sex** | **Sample timepoint (days post-positive test)** |
| --- | --- | --- | --- | --- | --- |
| CONV01 | G1m-1,3/G1m-1,3 | Mild | 21 | F | 8 |
| CONV02 | G1m-1,3/G1m-1,3 | Moderate | 69 | M | 32 |
| CONV03 | G1m-1,3/G1m-1,3 | Mild | 54 | F | 34 |
| CONV04 | G1m-1,3/G1m-1,3 | Mild | 55 | M | 34 |
| CONV05 | G1m-1,3/G1m1,17 | Mild | 63 | M | 32 |
| CONV06 | G1m-1,3/G1m-1,3 | Mild | 54 | F | 32 |
| CONV07 | G1m1,17/G1m1,17 | Moderate | 59 | M | 35 |
| CONV08 | G1m-1,3/G1m-1,3 | Mild | 61 | F | 35 |
| CONV09 | G1m-1,3/G1m-1,3 | Moderate | 52 | F | 24 |
| CONV10 | G1m-1,3/G1m-1,3 | Moderate | 22 | F | 37 |
| CONV11 | G1m-1,3/G1m1,17 | Mild | 54 | M | 24 |
| CONV12 | G1m-1,3/G1m-1,3 | Mild | 58 | F | 28 |
| CONV13 | G1m-1,3/G1m-1,3 | Moderate | 61 | F | 36 |
| CONV14 | G1m-1,3/G1m1,17 | Moderate | 60 | M | 36 |
| CONV15 | G1m-1,3/G1m-1,3 | Mild | 59 | M | 30 |
| CONV16 | G1m-1,3/G1m1,17 | Mild | 57 | F | 30 |
| CONV17 | G1m-1,3/G1m1,17 | Mild | 66 | M | 34 |
| CONV18 | G1m-1,3/G1m1,17 | Mild | 66 | M | 25 |
| CONV19 | G1m-1,3/G1m-1,3 | Mild | 65 | M | 36 |
| CONV20 | G1m1,17/G1m1,17 | Mild | 55 | M | 33 |
| CONV21 | G1m-1,3/G1m-1,3 | Mild | 50 | F | 30 |
| CONV22 | G1m-1,3/G1m-1,3 | Mild | 64 | M | 38 |
| CONV23 | G1m-1,3/G1m1,17 | Moderate | 58 | M | 23 |
| CONV24 | G1m1,17/G1m1,17 | Mild | 61 | M | 28 |
| CONV25 | G1m-1,3/G1m-1,3 | Moderate | 58 | M | 38 |
| CONV26 | G1m-1,3/G1m1,17 | Moderate | 59 | F | 28 |
| CONV27 | G1m1,17/G1m1,17 | Mild | 22 | F | 19 |
| CONV28 | G1m-1,3/G1m-1,3 | Mild | 49 | M | 41 |
| CONV29 | G1m1,17/G1m1,17 | Mild | 52 | M | 38 |
| CONV30 | G1m-1,3/G1m-1,3 | Moderate | 52 | F | 53 |
| CONV31 | G1m-1,3/G1m1,17 | Mild | 56 | M | 50 |
| CONV32 | G1m-1,3/G1m-1,3 | Moderate | 31 | M | 45 |
| CONV33 | G1m-1,3/G1m1,17 | Mild | 28 | F | 44 |
| CONV34 | G1m-1,3/G1m-1,3 | Mild | 24 | M | 38 |
| CONV35 | G1m-1,3/G1m-1,3 | Moderate | 52 | F | 45 |
| CONV36 | G1m-1,3/G1m1,17 | Moderate | 49 | F | 45 |
| CONV37 | G1m-1,3/G1m-1,3 | Mild | 56 | M | 56 |
| CONV38 | G1m-1,3/G1m-1,3 | Mild | 55 | F | 56 |
| CONV39 | G1m-1,3/G1m1,17 | Mild | 52 | M | 48 |
| CONV40 | G1m1,17/G1m1,17 | Mild | 24 | F | 48 |
| CONV41 | G1m1,17/G1m1,17 | Mild | 23 | M | 50 |
| CONV42 | G1m-1,3/G1m1,17 | Mild | 34 | F | 55 |
| CONV43 | G1m-1,3/G1m1,17 | Mild | 33 | M | 58 |
| CONV44 | G1m-1,3/G1m1,17 | Mild | 54 | M | 23 |
